# Are All Cells Created Equal? Novel Cell-Based Regenerative Therapies in Inflammatory Bowel Disease

**DOI:** 10.3390/ijms27052205

**Published:** 2026-02-26

**Authors:** Adam R. Peterson, Peter J. Eggenhuizen, Poh-Yi Gan, Charlotte Keung, Joshua Ooi, Gregory T. Moore, Rimma Goldberg

**Affiliations:** 1 Centre for Inflammatory Diseases, Department of Medicine, School of Clinical Sciences, Monash University, Clayton, VIC 3168, Australia; 2 Gastroenterology Department, Monash Health, Clayton, VIC 3168, Australia; 3 The Ritchie Centre, Hudson Institute of Medical Research, Clayton, VIC 3168, Australia

**Keywords:** inflammatory bowel disease, regenerative medicine, cell therapy, Crohn’s disease, ulcerative colitis, stem cells

## Abstract

Regenerative medicine, and in particular cell-based therapies, are under investigation as therapeutics in the management of inflammatory bowel disease, where despite significant advancements in management, prolonged remission is achieved in less than half of patients experiencing these disorders. In contrast to conventional immunomodulatory medications, these therapies are hypothesised to act through multiple pathways including via regenerative mechanisms, which may enable them to break through the current therapeutic ceiling. Potential therapy candidates include mesenchymal stem cells, human amnion epithelial cells, and regulatory T-cells, as well as their derivatives including extracellular vesicles. Extensive preclinical studies have demonstrated the multi-modal nature of these therapies as well as shared and unique properties. Controversy remains regarding contradictory study outcomes and the efficacy of regenerative therapies in human trials. In this narrative review, we first examine the mechanisms of these candidate cell therapies, including signalling via cytokines and extracellular vesicles, and interactions with immune cells, stromal cells, and the microbiome to determine differences and similarities between them. The second part delves into the current state of regenerative and cell-based therapy, focusing on mesenchymal stem cell, human amnion epithelial cell, T regulatory cells, and their respective extracellular vesicles in IBD treatment. Finally, we close by identifying the major literature gaps and barriers to bringing regenerative medicines to clinical use, resulting in recommendations for future research.

## 1. Introduction

The global burden of inflammatory bowel disease (IBD), including Crohn’s disease (CD) and ulcerative colitis (UC), has increased over time, and its health and economic impact continues to grow due to compounding prevalence. The burden of IBD in developing countries is low compared to Western nations, but it is expected to increase in line with industrialisation [1].

Current treatment strategies are almost entirely targeted at the aberrant immune response observed in IBD [2,3]. However, this focus has thus far yielded imperfect results. In contemporary pharmaceutical clinical trials, there is a therapeutic ceiling where deep and prolonged remission was achieved in less than half of patients [4,5], and the requirement for surgical management in UC and CD over long-term follow-up remains high with rates of 15–20% and 40–50%, respectively [6]. Furthermore, contemporary understanding of the varied response to the current therapeutic armamentarium is limited and there is a lack of available biomarkers to predict treatment response [7]. This therapeutic ceiling is being assessed by trials of combination advanced therapies [8]; however, this is not without risk, and serious infection [9] and possibly malignancy can be associated with current therapies especially when used in combination with thiopurine therapy [10].

A failure to achieve treatment targets means that patients with IBD continue to experience disproportionate rates of depression and anxiety [11], poorer quality of life [12], a higher likelihood of work disabilities and loss of productivity [13]. As a result of these complications active disease significantly increases direct healthcare costs [14].

Haematopoietic stem cell transplant (HSCT) is a medical procedure that involves the intravenous replacement of bone marrow with healthy blood forming cells in a patient with dysfunctional or depleted bone marrow and may be autologous or allogenous. It was the first cell-based therapy used in IBD and was noted to improve IBD when given for concurrent malignant indications but was limited by toxicity from conditioning regimes [15,16]. This has led to exploration of cell-based therapies, which were hoped to revolutionise the management of these complex diseases with improved and more durable outcomes through their ability to address multiple pathways beyond those inflammatory pathways targeted through traditional therapeutics.

Cell therapies and their derivatives have been extensively studied in the laboratory, and some have reached human clinical trials. The most advanced studies have occurred in haematopoietic stem cells and mesenchymal stem cells derived from the bone marrow, umbilical cord, and adipose tissue. However, other cell types present novel mechanisms and advantages that are now being assessed in a clinical environment.

The first part of this review will focus on the mechanisms of action of regenerative and cell-based medicine in IBD. Specifically, the role of cytokines, growth factors, and extracellular vesicles (EVs), the interaction with adaptive immune cells, particularly T regulatory cells, as well as innate immune cells, stromal cells, and the microbiome to elucidate the mechanisms of action of regenerative medicine for IBD. The second part of the review will focus on the current ‘state of play’ of regenerative and cell-based therapy. It will explore the development of haematopoietic stem cells, mesenchymal stem cells (MSCs), and human amnion epithelial cells (hAECs) and their use in IBD. The use of T regulatory cells (Treg) and EV will also be explored as a treatment for IBD.

## 2. MSC, hAEC and Treg Mechanisms of Action

**Figure 1:**
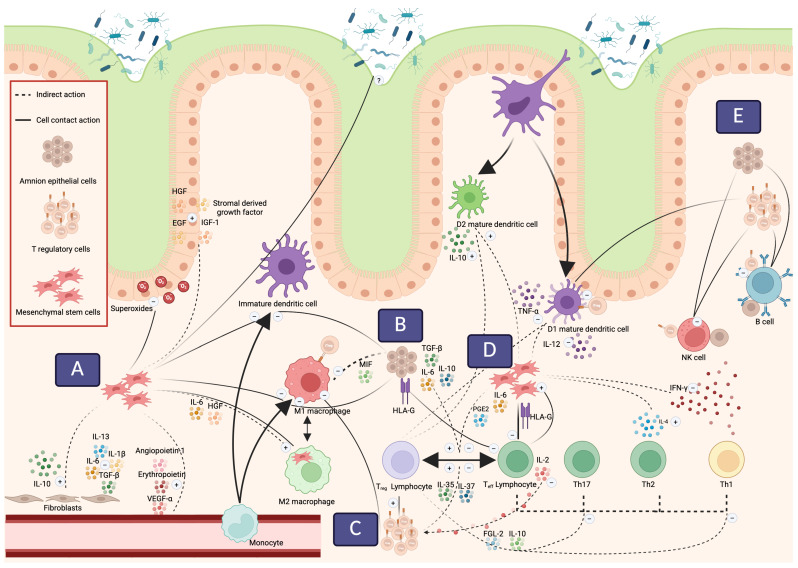
Mechanisms of cell therapies. (**A**) mesenchymal stem cells (MSCs) may directly affect the microbiome, polarise macrophages via efferocytosis, inhibit monocyte differentiation, and act as an antioxidant in epithelial disruption. Indirectly they may further mediate epithelial regeneration, angiogenesis and fibrosis. (**B**) Human amnion epithelial cells (hAECs) may directly inhibit monocyte differentiation, effector T cell proliferation via HLA-G and indirectly influence Treg/Teff proliferation and macrophage migration. (**C**) Regulatory T cells (Tregs) directly and indirectly inhibit Teff proliferation and activity, directly inhibit antigen presenting cell activation of Teff and indirectly polarise dendritic cells to tolerogenic subtypes. (**D**) Further action of MSC directs suppressive effects upon Teff via HLA-G, indirect inhibition of Teff whilst promoting Treg, promotion of tolerogenic dendritic cell activity and suppression of inflammation IFN-G from Th1 and NK cells whilst promoting IL-4 release from Th2 cells. (**E**) hAEC and Treg may both directly inhibit NK and B cells, whilst Treg may also directly inhibit antigen presenting dendritic cells. Created in BioRender. Peterson, A. (2025) https://BioRender.com/nx98r51 (accessed on 17 February 2026).

The progress of regenerative and cell-based medicine in IBD has been hampered by incomplete and sometimes conflicting evidence to explain the effects of these treatments. Further confounding the understanding of the actions of regenerative cell therapies are the various sources of cell therapies and culture conditions combined with differing actions depending on the tissue microenvironment to which they are delivered. The next section will explore the effects of cell-based regenerative therapies for IBD in terms of their role in cytokine and growth factor secretion, their ability to modulate adaptive and innate immune cells as well as their effects on stromal cells and the microbiome (Figure 1).

Initial in vitro studies demonstrated the differentiation of MSCs into mesenchymal tissues including osteogenic, adipogenic, and chondrogenic cells [17]. It was hypothesised that the administration of stem cells would result in differentiation of those cells and subsequent replacement of damaged tissue. This was supported by evidence of radiolabelled infused MSCs localising to the lung, liver, bone marrow and spleen as well as to tissue sites of injury in animal models [18,19]. Further studies using tissue PCR analysis found that human MSC may be found in murine tissues such as bone, cartilage, and muscle up to 13 months following infusion [20]. One of the first clinical applications, where sex-mismatched MSCs were administered in utero to a patient with osteogenesis imperfecta demonstrated bone engraftment via whole Y genome fluorescent in situ hybridisation at 9 months of age [21]. The same hypothesis was applied to the gut. MSCs have been observed to traffic to murine intestinal mucosa via endomicroscopy with labelled cells [22] and were possibly observed to differentiate to intestinal epithelial cells [23] or acquire epithelial properties in vitro [24]. However, MSC differentiation into non-mesenchymal cell lines is controversial and proponents argue that previously observed effects of MSCs may have been due to effects exclusive of cell differentiation [25] and there is a lack of evidence confirming engraftment in the intestine, particularly in human studies.

### 2.1. Role of Cytokines and Growth Factors

Contemporary evidence suggests a more important role for these cells in releasing cytokines, growth factors, peptides, as well as paracrine effects. This is supported by the observation of cell therapy effects persisting beyond the engraftment of cells [26].

Before the recognition of bone marrow MSCs, cytokine expression profiling was used in addition to cell surface proteins to identify mesenchymal progenitor cells. Cultured cells were observed to support haematopoiesis through the expression of granulocyte colony-stimulating factor, stem cell factor, leukaemia inhibitory factor, and macrophage colony-stimulating factor but interestingly also produced inflammatory cytokines IL-6 and IL-11. Co-culture with pro-inflammatory IL-1α increased expression of both haematopoietic mediators and inflammatory IL-6 and IL-11. Conversely, exposure to dexamethasone decreased the expression of IL-6 and IL-11 without effecting the other cytokines [27]. Following the identification of MSCs, isolated cells were found to produce similar cytokine and growth factor profiles [28]. These studies demonstrated the role of MSCs in supporting haematopoiesis in the bone marrow but also led to the hypotheses that MSC had a role in the regulation of inflammation and fibrosis in other tissues.

MSCs have been observed in vitro to modify the cytokine expression of innate and adaptive immune cells. Mature dendritic cells (DCs) type 1 had decreased secretion of TNF-α, whilst DC type 2 had increased IL-10 secretion. Natural killer (NK) and T_H_1 cells decreased interferon γ (IFN-γ) secretion, whilst T_H_2 increased secretion of IL-4. MSCs were noted to produce IL-6, IL-8, vascular endothelial growth factor (VEGF) and prostaglandin E2 (PGE2), and these levels increased with exposure to peripheral blood mononuclear cells (PBMCs). Interestingly, such immunomodulatory effects were diminished by PGE2 synthesis inhibitors [29]. IL-6 is produced by MSCs, and the anti-inflammatory effects mediated by PGE2 may depend on its production. This was demonstrated in a study of IL-6 knockout mice, where MSCs derived from those mice were less effective in suppressing T cell proliferation in a mixed lymphocyte reaction (MLR) assay [30].

Transforming growth factor β (TGF-β) is secreted by MSCs and is known to significantly alter immune responses through its effect on cell proliferation and differentiation. In vitro studies have shown a reduction in MSCs ability to decrease effector T cell differentiation when TGF-β is blocked with a monoclonal antibody [31]. Moreover, MSC may increase their secretion of TGF-β in inflammatory environments. This was shown in co-culture MSC experiments with both T_H_1-derived IFN-γ [32] and in T_H_2-activated murine model of asthma [33] and points the TGF-β as an important pathway in MSCs immune modulatory role in an inflammatory environment. Another study found that both IL-10 and TGF-β secretion was significantly enhanced in the presence of direct MSC to T-cell contact rather than cytokines exposure alone [34], which raises the question whether there are additional cell contact-mediated pathways that effect MSC function on T cells. However, in contradiction to these results, MSCs have been shown to inhibit TGF-β in studies of intestinal fibrosis, where TGF-β is known to be proliferative and pro-fibrogenic [35].

HAECs are cells with similar properties to MSCs that can be derived from the human placenta. HAECs have been demonstrated in vitro to secrete a similar cytokine profile to MSC with TGF-β, as well as IL-10, Il-6, TNF-α, fas ligand, TRAIL and macrophage migration-inhibitory factor [36]. Together, these factors are thought to explain hAEC’s immunomodulatory effect through suppression of T cells and B cells, and inhibition of macrophage migration [37].

Cytokines associated with regeneration have also been found to be secreted from MSCs including VEGF-α, IGF-1, epidermal growth factor, keratinocyte growth factor, angiopoietin-1, stromal derived growth factor-1, erythropoietin, and macrophage inflammatory protein-1α and β [38]. Similarly, hepatocyte growth factor (HGF) has been found to be produced by MSCs. HGF is involved in both angiogenesis and organogenesis and has been to shown to mediate regenerative effects of MSCs in experimental autoimmune encephalomyelitis [39]. This also appears to be a cell contact-mediated effect [34].

Treg are a CD25+, CD127–, forkhead box protein P3+ (FoxP3+) subset of T cells that are the primary mediators of peripheral immune tolerance. Treg differentiation and function has been the focus of a comprehensive review [40], which highlights the importance of both target cells and cytokines on Treg differentiation. Similarly, the therapeutic properties of Treg are both contact-dependent and non-contact-dependent, and probably have complementary action [41]. Cell surface co-inhibitory molecules such as programmed cell death 1 (PD-1) and cytotoxic-T-lymphocyte antigen 4 (CTLA-4) are important for Treg stability and function in vivo. Meanwhile, IL-10 and TGF-β are soluble factors required for Treg suppression of T effector function in murine colitis [42,43]. More recently IL-35, constitutively released by Treg, was found to suppress Teff cells which is in contrast to other members of the IL-12 heterodimeric family, IL-12 and IL-23 that have pro-inflammatory properties. Interestingly, recombinant IL-35 administration induced Treg differentiation, improved Treg/Teff ratio in colonic tissue, and ultimately reversed DSS colitis [44]. Another anti-inflammatory cytokine involved in enhancing Treg suppression is IL-37. Both IL-35 and IL-37 have been shown to be decreased in UC and CD serum compared to healthy controls [45] and have the capacity to modulate inflammation in murine models of IBD [46]. These studies provide further evidence of the importance of non-contact dependent Treg functions.

Foxp3+ Tregs specialise in suppressing specific Teff responses induced by specific transcription factors, and their function in response to these differs, including cytokine release [47]. For example, T-bet+ Treg inhibit DC production of IL-12 whilst inducing IL-10 via the co-inhibitory molecule TIGIT [48] as well as by secreting fibrinogen-like protein 2 (FGL-2) and IL-10 resulting in inhibition of TH1 and TH17 responses without altering TH2 responses [49]. An intriguing mechanism by which Treg may induce apoptosis is via IL-2 deprivation of Teff through the sequestering of IL-2 by upregulation of IL-2 receptor α-chain (CD25) [50]; however, given the important role of IL-2 in Treg homeostasis, it is unclear whether this is an essential mechanism in vivo, as discussed in this comprehensive review [51].

Whilst all three cell types, MSC, Treg and hAEC, have demonstrated effects mediated via cytokine signalling, these effects are often enhanced by cell-to-cell contact or only partially explain the observed phenomena suggesting a direct role between cell therapies and their targets can also be critical in their therapeutic effect. Therefore, it is likely cell contact-dependent and cell contact-independent mechanisms work synergistically to create the therapeutic effect in regenerative cell-based therapies for IBD.

### 2.2. Interaction with Adaptive Immune Cells

As with all cell and tissue-based transplants, consideration of immunogenicity in the use of these cell therapies has been examined. Both MSCs and hAECs do not provoke an allogenic T cell reaction despite low to intermediate expression of MHC class I and low levels of MHC class II. This may be due to the absence of co-stimulatory molecules including B7-1 and B7-2 on these cells [17,52], or constitutive mechanisms that may also account for their immunoregulatory role. In contrast, allogeneic Treg use is limited by host allo-rejection due to mechanisms including the expression of MHC class I, which has led to investigation of autologous therapies and preclinical evaluation in immunodeficient or adoptive immune model mice [53].

In vitro studies of MSC and hAEC cell-based therapy have demonstrated anti-proliferative effects on multiple cell types. Studies of MSCs in MLRs demonstrated antiproliferative effects that were independent of the MHC [54]. Using a transwell system, this antiproliferative effect has been observed and partially attributed to cytokines including TGF-β and HGF; however, further T-lymphocyte inhibition was observed with direct cell contact [31]. Importantly, this suppressive activity was not accounted for by alternate cytokine pathways including IL-10, PGE_2_ or tryptophan depletion [55]. Contrastingly, other investigators have found MSC dose-dependent T cell inhibition via PGE_2_ [56]. Lymphocyte suppression has also been observed following culture with amnion-derived MSC [57] and hAEC in C57BL/6 mice [37].

Treg may have anti-proliferative effects on Teff by acting as a sink for IL-2 via their high IL-2R receptor affinity resulting in depletion of local IL-2 constitutively necessary for Teff proliferation [58]. But in contrast to antiproliferative effects also seen with MSC and hAEC, Treg can have cytotoxic activity on Teff via cytolysis with granzyme A and perforin [59]. Additionally, granzyme B has been shown to be upregulated in Treg and Treg may induce cytolysis of B cells [60] and NK cells [61] via direct contact in a granzyme B and perforin-dependent manner. Additional direct contact actions of Treg possibly include cytolysis via the TRAIL-DR5 pathway [62] and the generation of pericellular adenosine via co-expression of the ectonucleotidases CD39 and CD73 [63,64]. Adenosine triphosphate (ATP) is primarily an intracellular molecule. Its presence outside the cell serves as a danger signal that activates immune and other cells through purinergic receptors, such as P2X7. The enzyme CD39 converts ATP to adenosine diphosphate and adenosine monophosphate (AMP), and subsequently, CD73 converts AMP to adenosine. Adenosine then engages immunoregulatory receptors like A2A [65]. Interestingly, CD39 and CD73 are also expressed on the surface of hAEC and may contribute to immune tolerance at the maternal foetal interface [66,67].

HLA-G antigens are membrane-bound and soluble, immunomodulatory factors in both innate and adaptive immune responses that were first identified in the placenta. HLA-G is present within intestinal biopsies of patients with CD and UC and may have a protective role [68]; furthermore, soluble HLA-G5 increases with levels of inflammation seen within the epithelium [69]. HLA-G protein expression has been observed in human MSCs and has been correlated with lymphoproliferative inhibition in a co-culture model of MSC/PBMC and phytohemagglutinin. HAECs have also been observed to produce membrane-bound and soluble HLA-G as well as HLA-E and pre-treating hAEC with anti-HLA-G and anti-HLA-E partially reverted their suppression of T cells in vitro [70]. These data raise a novel mechanism by which both MSC and hAEC may direct their immunoregulatory roles.

### 2.3. Interactions with T Regulatory Cells

Both MSCs and hAEC have been shown to increase the differentiation of naïve T cells to Treg in co-culturing conditions [29,36], but not through expansion of pre-existing Treg populations. This has been shown to be mediated through TGF-β secretion [71] and via induction of Foxp3 [72]. However, TGF-β independent mechanisms including MSC secreted extracellular vesicles have also mediated this differentiation [73]. MSCs programmed to produce IL-37 and CXCR4 in rat models of IBD showed enhanced MSC migration to the colon and MSC-dependent expansion of Treg and suppression of TH17 cells [74].

### 2.4. Interaction with Innate Immune Cells

Alongside interactions with T cells, MSCs interact with macrophages and cause polarisation to an immune-regulatory/anti-inflammatory phenotype (type 2). This has been proposed to occur via IL-6, HGF [75], and macrophage efferocytosis of experimental MSCs in a PGE_2_-dependent manner [56], similar to MSC interactions in models of asthma [76]. Additionally, they may actively inhibit differentiation of monocytes to either inflammatory macrophages or dendritic cells [77]. Similar results have been observed using hAEC in vitro [78] and models of interstitial lung disease, but this has not yet been observed in IBD models [79]. Efferocytosis may explain the effect of apoptotic and dead MSCs upon macrophage polarisation and perhaps is a rationale for why anti-inflammatory effects of MSCs and cell therapies persist beyond the presence of transplanted cells [80]. Umbilical cord MSCs treatment in DSS colitis has been shown to be mediated through interactions with neutrophils via inhibition of ERK phosphorylation [81] and umbilical cord MSCs inhibited expression of 15-lox-1 responsible for inflammatory responses of macrophages. Interestingly, this effect was proposed to be secondary to release of miRNA via EV release discussed below [82].

As may be expected, Tregs have also been observed to interact with antigen presenting cells including DCs required for the activation of Teff cells. Tregs attenuate DC activation of Teff through the co-stimulatory molecule CTLA-4 [83] and possibly through the induction of DC production of indoleamine 2,3-dioxygenase, which in turn induces pro-apoptotic molecules and Teff suppression through mechanism involving CTLA-4 and CD80 and/or CD86 [84]. Whilst the actions of Treg have been extensively studied in vivo in IBD, there is a paucity of examination of their specific interactions in IBD or IBD models following their transfer as a therapy.

### 2.5. Interactions with Intestinal Stromal Cells

Immune cell function relies on local signalling from mesenchymal and epithelial cells and regenerative medicine therapies have also been shown to directly interact with the intestinal stroma.

Inflammation of the colon mediated by immune cell dysregulation has been shown to increase production of reactive oxygen species that overwhelms endogenous anti-oxidant capacity leading to oxidative stress and epithelial cell disruption [85]. MSCs have been shown to prevent a reduction in glutathione (non-enzymatic antioxidant defences) and superoxide dismutase activity seen in DSS colitis suggesting an anti-oxidant role [86]. Interestingly, MSCs have also been shown to reverse a decrease in myenteric neuronal density and alteration in associated gene signatures seen in colitis, through antioxidant mechanisms potentially via MSC-derived superoxide dismutase.

MSCs have also been shown to interact with the stroma in decreasing intestinal fibrosis through the downregulation of profibrotic cytokines such as IL-1β, IL-6, IL-13 and, as previously discussed, TGF-β, with a resultant decrease in phosphorylation of Smad2 and Smad3, whilst IL-10 is upregulated [35]. Interestingly, in a TNBS rat model established fibrosis and epithelial-to-mesenchymal transition (EMT) was reversed [35]. Further studies have implicated HGF and tumour necrosis factor-stimulated gene 6 in limiting the progression of fibrosis by reducing activation of the smooth muscle cells and myofibroblasts in radiation-induced murine colitis [87].

### 2.6. Interactions with the Microbiome

The microbiome has become an area of intense research interest over the past decade, and of particular importance in the evaluation of IBD mechanisms. Yet there are limited studies examining interactions between gut bacteria and regenerative medicines. Fu, et al. found that following the treatment of rat TNBS colitis with human umbilical cord MSC, there was an improvement in healthy gut bacteria [88]. However, it remains unclear whether microbiome remodelling occurred due to a direct interaction of the MSC or because of the improved colitis.

## 3. Mechanisms of Extracellular Vesicles

Extracellular vesicles (EVs) are lipid bilayer-membrane vesicles containing proteins, nucleic acids, lipids and other metabolites that are released by a variety of cells, and have an established role in intercellular communication both in physiological and pathological settings [89]. EVs have been explored as biomarkers, potential therapeutic targets, and as drug delivery systems in IBD.

Studies into EVs have provided an explanation of some immunomodulatory and regenerative mechanisms of cell-based therapies [90] and led to further exploration of EVs as a therapy unto themselves. Contemporary evidence suggests that their therapeutic properties are mediated by horizontal transfer of mRNA, miRNA and protein.

However, EV content is not static and the local environment of the cells from which they are obtained including immediate intercellular neighbours and their activity, as well as the source of the cells themselves can alter their content [91,92]. This is highlighted in hAEC, where the gestational age of the cells may impact their efficacy [93]. Innovative researchers have harnessed these observations to further enhance the benefits derived from EVs [94].

Again, MSC-derived EVs are the most evaluated and they have been found to mediate many of the effects seen in these cells and may possibly account for those observations. This includes polarising active CD4^+^ T cells to Treg via an EV treated THP-1 cell line [73]. However, novel and independent mechanisms of EVs have also been suggested.

EV mechanisms have been evaluated both without knowledge of their cargo and with specific cargo in mind or even loaded into vesicles. EVs derived from MSC of varying sources and hAEC have been shown to alleviate colitis in murine DSS models [95,96]. Multiple mechanisms have been suggested including via ubiquitin modification level [97] and macrophage polarisation [98,99]. A recent study by Tolomeo and colleagues found that in contrast to previous evidence discussed here both naïve and induced murine MSCs worsened colitis, and yet their EVs still promoted healing [100]. In other disease models, MSC EVs have been shown to regulate T cell polarisation [73], dendritic cell differentiation [101], inhibit macrophage activation, and prevent oxidative injury [102], which may hold relevance to their effect in IBD.

mRNA and miRNA have been of particular interest and explored across a range of regenerative medicine applications as a potential mechanism of EVs. IGF-1R mRNA has been shown to sensitise renal tubular epithelial cells to the beneficial effects of IGF-1 in acute kidney injury [103] and miR-17-92 carrying EVs provide a regenerative effect in neuronal axonal growth [104]. Other miRNA have been shown to inhibit cancer growth [105] and promote angiogenesis via miR-125 transfer to endothelial cells providing an alternate explanation of tissue repair [106].

mRNA have also been implicated in homeostasis of the intestinal epithelial barrier, which is impaired in IBD. MSC EVs have a protective effect on the intestinal epithelial barrier, improving intestinal permeability and preserving goblet cells [107] and specifically miR-34a-5p may improve intestinal barrier function through epigenetic modification in ischaemia/reperfusion injury of the gut [108].

MSC and MSC-derived EVs have been shown to ameliorate inflammation in models of IBD via inhibition of the inflammasome, reduction in activation of caspases and a resultant decrease in pyroptosis. This observation is proposed to be mediated via miRNA in EVs. miR-378a-5p, miR-203a-3p.2 and miR-539-5p have been implicated in the EV mediated improvement of DSS colitis via reduction in inflammasome activation, caspase inhibition and reduced pyroptosis. Cai, et al. found that EVs carrying miR-378a-5p inhibited inflammasome activation, Caspase-1 cleavage and reduced pyroptosis [109], whilst Xu, et al. found that miR-203a-3p2 reduced Caspase-4 expression with a resultant reduction in macrophage pyroptosis. [110]. Wang, et al. identified miR-539-5p to inhibit NLRP3 and Caspase-1 [111].

Of intense interest in relation to IBD was the finding that miR-200b inhibits epithelial–mesenchymal transition (EMT) through TGF-β [112]. EMT is a process whereby polarised epithelial cells undergo multiple biochemical changes to become a mesenchymal cell phenotype with the ability to migrate and produce extracellular matrix components with a decreased tendency toward apoptosis, which, in a physiological state, aids in healing. However, this process is associated with fibrosis in pathological states, including IBD [113]. miR-200b has been found to be reduced in IBD and it inhibits TGF-β1 EMT [112]. Subsequently, bone marrow MSC-derived EVs were transfected with lentivirus to overexpress miR-200b and decreased EMT and intestinal fibrosis in a TNBS rat model [114]. Other groups have found that umbilical cord MSC-derived EVs containing miR-21, miR-23a, miR-125b, and miR-145 suppress myofibroblast formation through TGF-β/SMAD2 and may reduce fibrosis independent of EMT [115]. Conventional IBD therapies do not influence EMT or significantly alter fibrosis making this finding of particular importance.

## 4. State of Regenerative and Cell-Based Therapy

### 4.1. Haematopoietic Stem Cells

HSCT has been studied in IBD for over 20 years. Initially it was hypothesised that HSCT may result in the abolition of ‘active’ T cells or immune memory, providing long term disease remission [15,16]. Subsequent interventional studies of autologous HSCT in CD demonstrated an excellent initial response but diminished long-term, medication-free remission rates [116,117] and there was subsequent conjecture as to whether mobilisation with cyclophosphamide and anti-thymocyte globulin or the HSCT itself provides clinical benefit. This was addressed with the me-controlled autologous stem-cell transplantation in treatment refractory Crohn’s (ASTIC) trial [118], which failed to demonstrate a significant difference between mobilisation and autologous HSCT or mobilisation and conventional therapy in its ambitious primary outcome of sustained clinical remission, without endoscopic activity of disease and the ability to stop conventional therapy. Whilst exploratory analyses suggested this therapy may be beneficial in selected patients, a high rate of treatment associated adverse events precluded their widespread adoption [119]. More recently, the Autologous Stem cell Transplantation in refractory Crohn’s disease—Low Intensity Therapy Evaluation ASTIClite trial assessed an attenuated mobilisation and conditioning regime versus placebo in a more conventional primary outcome of week 48 endoscopic remission. This trial was terminated early after all patients in the intervention group experienced serious adverse events and one died [120]. These same concerns have limited the extension and use of allogeneic HSCT, in which adverse events would be expected to be higher due to graft versus host disease. A randomised controlled trial protocol of non-myeloablative allogenic HSCT in refractory CD [NCT01288053] reported one death and was terminated without publication. Despite these findings, autologous HSCT continues to be offered in some centres, particularly in patients with the severest of disease phenotypes [121] including monogenic IBD [122].

### 4.2. Mesenchymal Stem Cells

**Figure 2:**
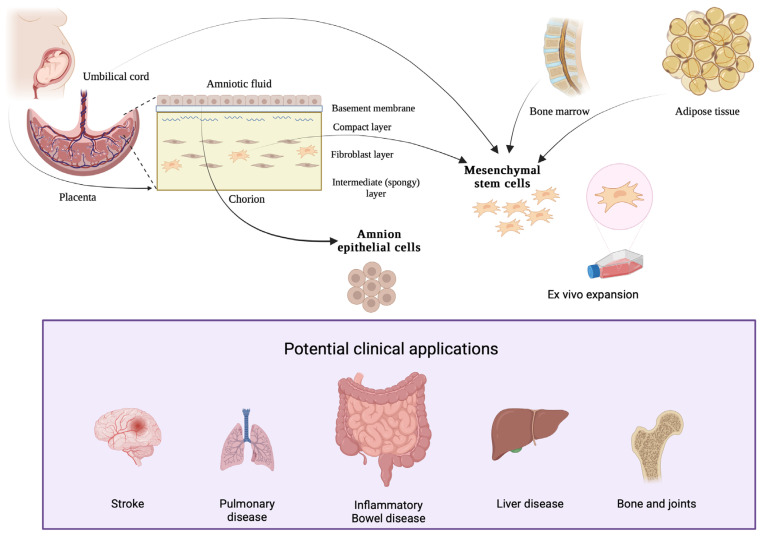
Isolation and production of MSC and hAEC. MSCs are frequently isolated from bone marrow, placenta, umbilical cord, and adipose tissue. They require in vitro expansion due to the low numbers that can be isolated. In contrast hAEC isolated from the placenta does not require expansion. Both cell types have been investigated in multiple inflammatory conditions including IBD. Created in BioRender. Peterson, A. (2025) https://BioRender.com/dnyofpc (accessed on 17 February 2026).

Whilst bone marrow-derived cells had been investigated in animal models of IBD since the 1990s, isolated MSCs were first applied to a rat 2,4,6- trinitrobenzene (TNBS) model of colitis in 2008, where they demonstrated accelerated healing of colitis [123]. Concurrently, MSCs with similar properties were isolated from lipoaspirates [124] and utilised via local injection in phase I trials for perianal fistulising CD, where they have been administered within and adjacent to fistulae by colorectal surgeons at the time of examination under anaesthesia [125]. Whilst bone-marrow derived MSCs have been studied in perianal fistulising CD [126,127], more studies have examined adipose derived MSCs in both autologous and allogeneic forms [128] due to easier and more patient-acceptable harvesting techniques (Figure 2). Other sources include the umbilical cord and placenta. The ADMIRE-CD phase 3 study using locally injected human adipose MSCs showed a significant improvement in inducing remission of perianal fistulising CD [129] and both safety and efficacy were maintained in these patients at 52 weeks on follow up [130]. However, a follow-up phase III study, ADMIRE-CD II, failed to demonstrate an improvement in fistula closure compared with placebo [131]. The commercial product, darvadstrocel (Alofisel, Takeda), which was initially approved for treatment of complex perianal fistulas via local injection and was available in Europe and Japan has since been withdrawn from market.

Concerns regarding the intravenous (IV) administration of MSCs arose from preclinical studies including their availability in the target tissue, pulmonary sequestration and potential tumorigenicity [132]. Nonetheless, there have been investigations into IV administration for refractory luminal disease in humans that demonstrated initial safety and feasibility using bone marrow-derived MSC [133,134]. Unfortunately, the only phase III study of IV MSC therapy was discontinued due to perceived trial design flaws [135]. A second trial is listed as completed at clinicaltrials.gov (NCT00482092), but no publication is available. More recently, the same product used via colonoscopy-delivered injection for refractory colonic CD [136] and UC [137] showed preliminary feasibility, safety, and efficacy.

Separate investigation into the safety and efficacy of IV-administered placental-derived mesenchymal-like adherent stromal cells (MLASCs) demonstrated short-term safety and feasibility in CD [138].

Most ongoing preclinical and early clinical trials in regenerative medicine targeted at IBD use MSC and their derivatives. Despite this, their clinical use has until recently been restricted to refractory perianal fistulising CD and at present there are no approved MSC-based therapies for IBD available outside of clinical trials.

Due to the high costs previously associated with the use of Darvadstrocel and limited availability, some centres have adopted the use of simple preparations including platelet rich plasma, microfragmented adipose tissue, and lipoaspirates, thought to contain MSCs, and injected these around fistulas. This has been shown to be effective in uncontrolled trials [139]. However, high-quality evidence from randomised controlled trials is lacking. Furthermore, the use of undifferentiated tissue raises problems with quality control of such techniques, their reproducibility and their applicability. Microfragmented adipose tissue products and unregulated MSC products remain available to patients, particularly in the United States [140].

### 4.3. Amnion Epithelial Cells

The amniotic membrane from which MLASC and hAEC are derived (Figure 2) has been used in medical practice throughout the 20th century for a variety of surgical procedures [141]. Amniotic membrane transplant continues to be utilised in corneal and conjunctival reconstructive procedures as a basement membrane or temporary graft deposition [142]. hAEC are a desirable cell for regenerative medicine due to their availability, stability of their karyotype, and low risk of allogenic rejection [143]. In contrast to MSC, they are able to be obtained in sufficient quantities so that in vitro expansion is not required and there has been no evidence of the formation of tumours in vivo [144]. hAEC and their conditioned media have been studied in dextran sodium sulfate (DSS) and TNBS models of acute and chronic murine colitis [96,145]. More recently, a first-in-human phase I study of locally injected hAEC in perianal fistulising CD demonstrated safety and feasibility [146]. However, the use of hAEC in IBD remains limited to clinical trials.

### 4.4. Regulatory T Cells

Due to their effects, particularly on T effector lymphocytes, Tregs have been implicated in multiple inflammatory diseases particularly those dependent on T cell-mediated inflammation, including IBD [147]. There is conflicting evidence as to whether Tregs are truly dysfunctional in IBD or whether T effector cells may be resistant to their effects in IBD pathogenesis, as has been previously reviewed [148].

**Figure 3:**
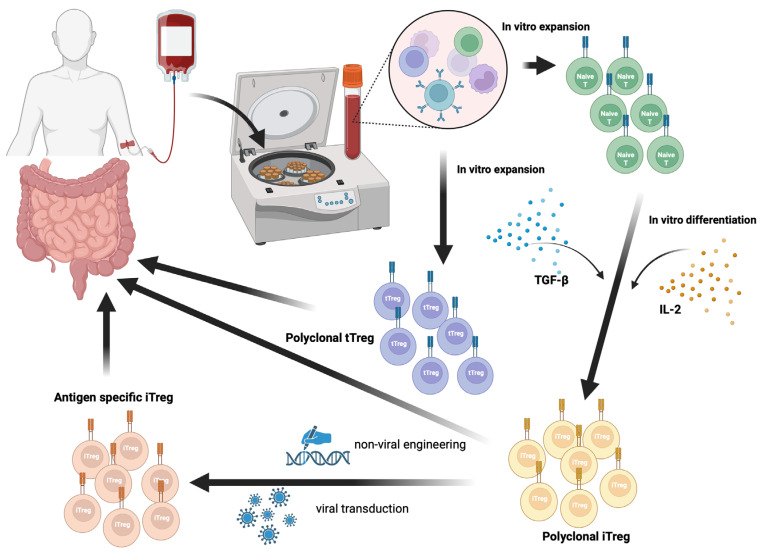
Types of Treg therapy in IBD. Polyclonal tTreg may be isolated in low numbers from PBMCs, whilst polyclonal iTreg may be induced from naïve T cells using TGF-β and IL-2. More recently engineered iTreg have been created using novel viral transduction and CRISPR technology and investigated as therapeutics in inflammatory conditions including IBD. Created in BioRender. Peterson, A. (2025) https://BioRender.com/mv2dxcp (accessed on 17 February 2026).

Irrespective of their role in the pathogenesis of IBD, Tregs have been investigated as a potential cellular therapy due to their ability to suppress immune responses and induce a more tolerogenic local tissue environment. There are two major subtypes of Tregs: those that mature in the thymus (tTreg) and those T cells that are induced to a regulatory phenotype in the local tissue (iTreg) (Figure 3). Polyclonal Tregs have been used in therapeutic trials for inflammatory conditions including type I diabetes, where it was shown that the transferred Treg were safe, maintained their suppressor phenotype and persisted for up to a year in patients [149]. In IBD, the TRIBUTE trial (NCT NCT03185000) is currently exploring in vitro-expanded autologous polyclonal Tregs for CD [150] and autologous Treg were explored in a single patient with treatment resistant UC [151]. A phase I/II trial of chicken ovalbumin (OVA)-specific Treg (Ovasave) in IBD showed they were well tolerated, and exhibited a dose-dependent efficacy with 40% of patients experiencing a reduction in CD activity up to 8 weeks post transfer [152]. However, this study did not progress to phase III due to challenges in the in vitro cell manufacture process. Further studies have examined the use of antigen-specific Treg therapies with the hypothesis that they may provide enhanced targeted immunosuppression, possibly leading to less risk of global immunosuppression. Antigen specificity has been achieved using chimeric antigen receptors (CAR) [153,154] and T cell receptors (TCR) [155]. Interestingly, autologous CD19 CAR T effector cells have recently been successfully used in a single patient with treatment-resistant UC highlighting further possibilities of antigen targeted therapies [156]. In Treg therapies, preclinical studies have been successful in ameliorating murine models of colitis, but whilst clinical trials of antigen specific Treg are underway in haematological disease and organ transplant, there have not been studies in human IBD. Overall, advancements in Treg cell therapies have shown the technology is an innovative way of approaching challenges faced by immune dysregulation in many diseases, including IBD.

### 4.5. Extracellular Vesicles

The role of EVs as therapeutics has been an area of intense interest for the past decade and EVs produced by stem cells and tolerogenic immune cells including Tregs and M2 macrophages may hold the most promise as they echo their originator cell immunomodulatory properties [157]. EVs are a commercially attractive alternative to whole cell regenerative medicines as they circumvent many limitations of cell therapy including the need for expansion as well as specialty storage and transport.

Studies of EVs derived from MSCs of heterogenous origin including human umbilical cord, placenta, adipose tissue and marrow, as well as murine marrow, adipose tissue and olfactory sources have demonstrated their efficacy in murine models of IBD (Table 1). Similarly, hAEC-derived conditioned media containing EVs had similar outcomes to hAEC treatment in improving clinical scores in a DSS model of colitis [96].

Preclinical studies of EVs isolated from other immune cells have also shown promise. EVs derived from M2b macrophages have been shown to attenuate DSS colitis [157]. EVs derived from Treg have been implicated in their tolerogenic role through in vitro studies and murine Treg derived EVs have demonstrated efficacy in murine models [158,159]; however, these studies have not been replicated using human Treg and at this time no human studies have been completed.

Despite MSC-EVs having been studied in human steroid refractory graft versus host disease [160] and chronic kidney disease [161], there are no in-human studies of IBD. This may be due to the significant heterogeneity of MSC-EVs as a therapeutic due to the variety of tissues from which MSCs are isolated, combined with their subsequent culture conditions and the way EVs are then produced. Our group is conducting a phase I trial of hAEC-derived EVs in human perianal fistulising CD (ACTRN12624000778583).

**Table 1:** Studies of MSC-derived EVs.

Trial	Cell Type	EV Isolation Method	Model	Outcomes
Barnhoorn et al., 2020 [162]	Murine bone marrow-derived MSCs	Ultracentrifugation	Murine Dextran Sulfate Sodium (DSS)	Decreased disease activity index (DAI), prevent weight loss, prevent colon shortening. Improved histological scores. Decreased proinflammatory cytokines. Improved histological score. Reduced cleaved caspase-3.
Cai et al., 2021 [109]	Human umbilical cord-derived MSC	Ultracentrifugation, filtration, precipitation	Murine DSS	Decreased disease activity index (DAI), prevent weight loss, prevent colon shortening. Improved histological scores. Decreased proinflammatory cytokines. Inhibit NLRP3 inflammasomes in macrophages and delay cell proptosis
Cao et al., 2019 [163]	Murine bone marrow-derived MSCs	Ultracentrifugation	Murine DSS	Decrease DAI. Promoted M2-like macrophage polarisation. Decreased proinflammatory cytokines. Potentially mediated by the JAK1/STAT1/STAT6 signalling pathway.
Duan et al., 2020 [164]	Human placental-derived MSCs	Ultracentrifugation, filtration	Murine trinitrobenzene sulfonic acid (TNBS)	Decreased disease activity index (DAI), prevent weight loss, prevent colon shortening. Improved histological scores. Reduced intestinal inflammation and oxidative stress.
El-Desoky et al., 2022 [165]	Rat bone marrow-derived MSCs	Ultracentrifugation	Rat acetic acid	Normalisation of histopathological features of colitis.
Heidari et al., 2021 [166]	Murine adipose-derived MSCs	EXOCIB kit (Cib biotech) bead isolation	Murine DSS	Decreased disease activity index (DAI), prevent weight loss, prevent colon shortening. Improved histological scores. Decreased MPO and pro-inflammatory cytokines. Increased percentage of Treg cells in spleen.
Li et al., 2020 [167]	Human adipose-derived MSCs	Ultracentrifugation, filtration	Murine DSS	Decreased disease activity index (DAI), prevent weight loss, prevent colon shortening. Reduced inflammatory cytokines. Phosphorylated and total JNK1/2, STAT3, JAK1/2 protein levels were reduced. Equivalence to MSC.
Liu et al., 2019 [98]	Human bone marrow-derived MSCs	Ultracentrifugation	Murine DSS, murine TNBS	Decrease DAI, prevent weight loss, prevent colon shortening. Improved histological score. Decreased MPO. Decreased proinflammatory cytokines. Demonstrated that action is macrophage-dependent.
Ma et al., 2019 [168]	Human umbilical cord-derived MSC	Ultracentrifugation, filtration	DSS	Decrease DAI, prevent weight loss, prevent colon shortening. Improved histological score. Decrease pro-inflammatory cytokines.
Mao et al., 2017 [95]	Human umbilical cord-derived MSC	Ultracentrifugation, filtration	Murine DSS	Decrease DAI, prevent weight loss, prevent colon shortening. Improved histological score. Decreased proinflammatory cytokines. Increased IL-10.
Tian et al., 2020 [169]	Murine olfactory ecto-mesenchymal stem cells	Ultracentrifugation	Murine DSS	Decrease DAI, prevent colon shortening, improved histological score. Inhibit differentiation of Th1/Th17 cells, promote Treg induction.

## 5. Regenerative Medicine Interactions in IBD: Summary and Future Directions

Here, we have explored the development, mechanisms and use of HSC, MSC, hAEC, Treg and their respective EV as regenerative therapies for IBD. A tremendous breadth of possible mechanisms and interactions have been suggested and whilst the literature is dominated by evaluation of MSCs from various sources there are significant overlapping properties of MSC with hAEC and Treg. As both UC and CD are likely mediated via multiple pathogenic pathways [170], this feature of cell-based therapies makes them highly attractive as a novel treatment in IBD.

However, there is an inherent heterogeneity in cell-based products due to donor variability [171,172], which is reminiscent of the challenges faced by those seeking to alter the microbiome and treat IBD through faecal microbiota transplantation [173]. Additionally, the dynamic nature of cell-based therapies, their derivatives and the effect their local environment has on their function need to be considered. Whilst it is possible they may act simultaneously through the varied mechanisms we have discussed, it seems more probable that their exact mechanisms will vary and change depending on their donor source, expansion, storage and the tissue to which they are delivered. This may further explain some of the differences observed in cell function for example the production of TGF-β in inflammatory versus fibrotic environments.

Despite an intense interest in regenerative medicine, extensive pre-clinical evaluation of multiple candidate therapies and now burgeoning clinical studies, regenerative medicine remains limited to the local treatment of perianal CD in only specific regions. There remain significant obstacles to scaling and increasing the availability of cell-based medicine. Few centres have the expertise or facilities to provide highly complex Good Manufacturing Practice compliant procurement, expansion, preparation or even storage and these processes leave cell-based therapies as highly expensive treatments [174].

EVs may hold promise in overcoming these obstacles and working toward a shelf ready product, but at present they remain highly costly to produce. Furthermore, strategies to overcome donor variability such as immortalisation of cell lines to produce more standardised EVs create further difficulties. Concerns remain for safety of immortalised products and their risk of tumorigenicity and while necessary, the current regulatory requirements for these products significantly increase research costs [175], which in turn may hamper progression of cell therapies to human trials.

Multiple groups have proposed manners in which cell and cell-based therapies may be ‘standardised’ [176] and this may lead to more comparable outcomes between studies, groups and research centres. A particular focus on accurate assessment of EV cargo through the wider availability of RNA sequencing may enable comparisons between studies and sources and furthermore lead to optimisation of EV treatments. More consistent use of comparator groups and accurate descriptions of EV content may have the added benefit of aiding the navigation of complex regulatory framework across multiple geographic areas.

While regenerative medicine may hold promise in the field of IBD, its potential is yet to be realised. Ongoing studies to understand the multitude of both shared and individualised pathways by which different cell-based therapies act may enable increased progression to clinical trials and ultimately highly valuable treatments for this population.

## Data Availability

The original contributions presented in this study are included in the article. Further inquiries can be directed to the corresponding author.

## Abbreviations

The following abbreviations are used in this manuscript:

AMP	Adenosine monophosphate
ATP	Adenosine triphosphate
CAR	Chimeric antigen receptor
CD	Crohn’s disease
DAI	Disease activity index
DC	Dendritic cell
DSS	Dextran sodium sulfate
EMT	Epithelial–mesenchymal transition
HSCT	Haematopoietic stem cell transplantation
hAEC	Human amnion epithelial cell
HGF	Hepatocyte growth factor
IBD	Inflammatory bowel disease
IFN-γ	Interferon γ
IGF-1	Insulin like growth factor 1
iTreg	Induced T regulatory cell
MLRs	Mixed lymphocytes reactions
MLASC	Placental-derived mesenchymal-like adherent stromal cell
MSC	Mesenchymal stem cell
NK	Natural killer cell
PBMC	Peripheral blood mononuclear cell
PGE2	Prostaglandin E2
TCR	T cell receptor
TNBS	2,4,6-trinitrobenzene sulfonic acid
TNF-α	Tumour necrosis factor alpha
TGF-β	Transforming growth factor β
TRAIL	Tumour necrosis factor-related apoptosis-inducing ligand
Treg	T regulatory cells
tTreg	Thymic T regulatory cell
UC	Ulcerative colitis
VEGF	Vascular endothelial growth factor
